# Classification of thoracic spine fractures: the four-column theory

**DOI:** 10.1007/s00264-023-05778-x

**Published:** 2023-03-21

**Authors:** Dakheel A. Aldakheel

**Affiliations:** 1 Colleg of Medicine, Imam Abdulrahman Bin Faisal University, Dammam, Saudi Arabia; 2 Department of Orthopaedic Surgery, King Fahd Hospital of the University, Khobar, Saudi Arabia

**Keywords:** Thoracic spine, Fractures, Spine instability, Rib cage, Sternum, The fourth column

## Abstract

**Purpose:**

The purpose of this study is to present a classification of thoracic spine fractures based on anatomical and biomechanical characteristics.

**Methods:**

This is a narrative review of the literature.

**Results:**

The classification is based on the relationship between movement and common forces acting on the spine. A mechanistic concept is incorporated into the classification, which considers both movements and the application of forces, leading to pathomorphological characteristics. A hierarchical ranking determines the severity of fractures within the thoracic spine, and treatment recommendations are presented in each category. The fourth column of the spine is incorporated into the classification through direct and indirect mechanisms.

**Conclusions:**

The proposed classification accommodates several advantages, such as simplicity and practicality, that make this classification helpful in daily practice. The dynamic relationship between movement and force provides a better understanding of the fracture mechanism. Finally, incorporating the fourth column will strengthen the indication for surgical management. To the best of our knowledge, this classification is the first classification developed uniquely for the thoracic spine fractures and will help to address a critical gap in the literature.

## Introduction

The thoracic spine fractures are underrepresented in the literature, and most publications focus on the thoracolumbar spine (T11-L2). As far as the thoracic spine is concerned, no unique fracture classification has been reported. There are no clear surgical indications for thoracic spine fractures; however, instability, malalignment, and neurological status are considered for surgical intervention [1].

Spine instability has been defined under physiological loads [2], which might be imprecise due to the lack of clinical and radiological correlation [3]. However, abnormal and increased movement can result in spine instability when spine stiffness is lost [4]. In spite of the biomechanical challenges associated with testing thoracic spine stiffness, recent research has added valuable information that can be utilised to develop a classification specific to the thoracic spine.

Bohler was the first to attempt to classify spine fractures in 1929, developing a classification for the thoracolumbar spine fractures [5]. In the following years, spine fractures were classified as stable or unstable based on their ability to increase deformity [6]. A concept relevant to further research has been developed: vertebral columnar injury [7]. Later, the three-column theory was introduced, suggesting that acute instability arises only when two out of three columns are involved [8]. Numerous classifications have been reported since the 1980s and have focused on the thoracolumbar spine [9–12], and thoracic spine fractures remain understudied.

A classification is grouping events or objects with common characteristics. It serves as a common language or predicts the behaviour of its member. A classification per se is a generalization, and the nature of a classification is to lose details to favour grouping. Lacking details can be acceptable if the classification provides valuable information [13]. The ideal spine classification remains challenging since there is no minimum content requirement [14]. However, a classification should be clinically relevant, simplified for daily use, limited numbers of categories, and has a prognostic value for treatment recommendation.

For a holistic approach to be realised, the hypothesis is that acknowledging key anatomical characteristics and recent biomechanical evidence will lead to the realisation that a more comprehensive approach to thoracic spine traumatic fractures can be adopted.

## Concept of thoracic spine classification

This classification is an evolution of Holdsworth’s original work [15]. His classification is based on two-column model, adapting movement as a mechanism of injury. Although he acknowledged the importance of the rib cage for the stiffness of the thoracic spine, it was not considered in the classification. His classification model was advocated for the whole spine.

This paper presents a classification based on four columns concept, of which the sternal-rib complex, as Berg [16] advocated, is considered the fourth column of the thoracic spine. Moreover, the posterior column has been redefined as the posterior bone complex to suit its unique characteristics as the posterior thoracic spine. The anterior and middle columns are defined as advocated by Denis [8].

The classification is based on the relationship between movement and common forces. The primary deforming factor is movement, whereas common forces, which include compression, distraction, and torsion forces, are considered the main categories. Type A injuries occur due to axial compression, which commonly affects one or two vertebral columns. Type A compression injury is furtherly divided based on movement, which will determine where and how the vertebral columns are affected. Type B represents a dynamic relationship between the primarily tensile force acting posteriorly and a simultaneous secondary compression force acting on the anterior vertebral column. Type C results from torsional injury acting axially either in flexion or extension. The hierarchy arrangement within the subcategories is devised to reflect ongoing events of force and movement.

A summary of fracture classification, fracture characteristics, and instability suggestions are given in Table 1.

**Table 1:** Thoracic spine fracture classification: main categories, subdivisions, fracture morphology, and status of stability

	Fracture characteristics	Status of stability
Compression-flexion(A1)		
A.1.1	Vertebral plate injury	Stable
A1.2	Vertebral body burst fracture	Stable if no instability of 4th column
A1.3	Vertebral body wedge fracture	Stable if no instability of 4th column
A1.4	Teardrops superior vertebral body	Stable if no instability of 4th column
Compression-extension (A2)		
A2.1	Posterior spinous process/lamina/facet joint	Stable
A2.2	Costovertebral joint fracture unilateral/bilateral	Stable if no instability of 4th column
A2.3	Teardrop vertebral injury/anterior longitudinal ligament injury	Potential instability
A2.4	Discal injury ± anterior vertebral body fracture	Unstable
Distraction injury (B)		
B1	Spinous process fractures/interspinous ligament injury	Stable
B2	Laminar fracture/facet joint injury	Stable if no instability of 4th column
B3	Costovertebral joint fracture and pedicle fracture	Potentially unstable
B4	Discal extension	Unstable
Rotational flexion (C1)		
C1.1	Fracture of the posterior corner of middle column / pedicle fracture	Stable if no instability of 4th column
C1.2	Fractures of pedicles over multiple levels	Potentially unstable
C1.3	Fracture of vertebral body	Unstable
C1.4	Lateral translation of the vertebral column	Unstable
Rotational extension (C2)		
C2.1	Fracture of spinous process	Stable
C2.2	Costovertebral joint and lamina fracture + / − facet joint	Potentially unstable
C2.3	Pedicle fracture and discal extension, no vertebral translation	Unstable
C2.4	Posterior translation of the vertebral column	Unstable

The following section discusses categories and subcategories and provides instability suggestions and treatment recommendations.

## Compression injury (Type A)

### Compression-flexion injury (Type A.1)

A fracture pathoanatomical feature, in addition to compression, is flexion. The amount of flexion will determine where the force will be applied to the vertebral body. Characteristically, it is a bi-columnar injury, mainly affecting the anterior and middle columns. The teardrop fracture is allocated at the bottom because of two reasons. First, the mechanism is complex and involves a coupling movement. A coupling movement of flexion and forward translation of the cephalic vertebra is expected to yield this injury pattern. Secondly, in hyperflexion, indirect injury of the fourth column is likely to occur, representing an unstable environment.

#### Type A1.1

Essentially, this type of injury involves an impaction of the vertebral plate. Because of natural thoracic kyphosis, no flexion motion is to be expected. The central part of the vertebral plate is involved affecting both the anterior and the middle columns (Fig. 1.1). Typically, the posterior structures are unaffected, and spinal canal narrowing does not occur. The vertebral height is not reduced, and neurological injury is not expected. For that, this injury is stable and treated conservatively.

**Fig. 1:**
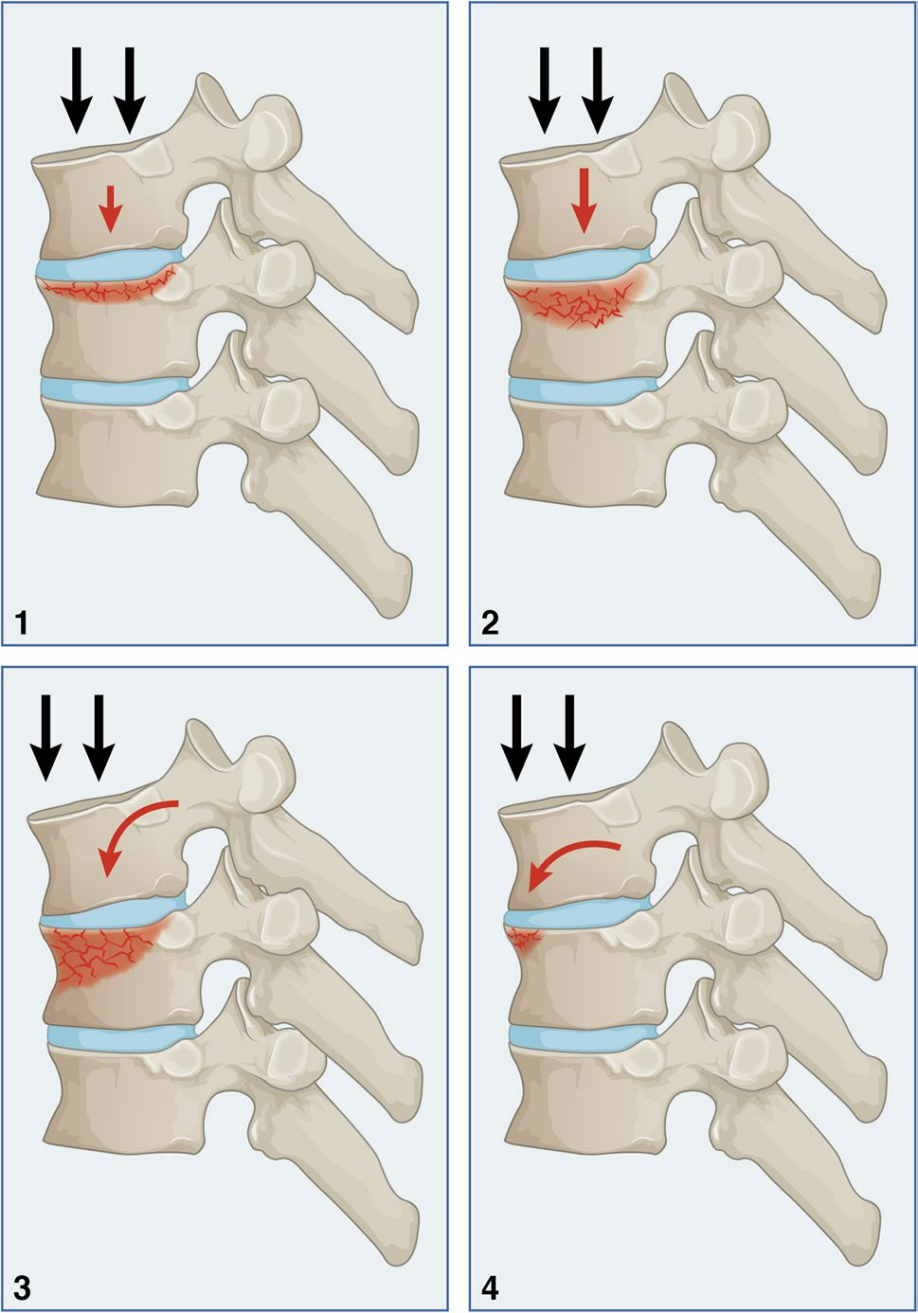
Compression-flexion injury (Type A1)

#### Type A 1.2

The middle and anterior columns are compressed more axially with mild flexion, resulting in a burst fracture (Fig. 1.2). As a result of the intact costovertebral complex, the vertebral height is minimally reduced, and boney retropulsion is minimal. Consequently, neurological injury is not expected. This is a stable facture and can be treated conservatively.

#### Type A 1.3

In moderate flexion, axial compression is concentrated on the anterior column, with a lesser degree on the middle column. The vertebral height is moderately reduced, affecting more the anterior column, and less than 50% wedging may be seen. Bony retroversion into the spinal canal would be expected to be mild because of intact both facet joint and costovertebral complex. The fracture can be treated conservatively and is stable.

#### Type A1.4

This injury occurs due to hyperflexion coupled with forward translation leading to a teardrop fracture of the superior anterior corner of the anterior column (Fig. 1.4). The size of the teardrop lesion is less than 10% of the anterior vertebral height. The bony lesion can be seen as a corner fracture of the vertebral body or a separated bony chip that can be appreciated in computer tomography. The middle column and the posterior structures are not affected. The overall vertebral height and spinal canal are maintained. A careful evaluation of the fourth column is required because of the indirect mechanism of injury, which renders potential instability. This type of injury can be treated conservatively without a fourth-column instability.

### Compression-extension injury (Type A2)

An extension is a deforming movement that is primarily affecting the posterior column. Vertical compression occurs first, followed by bending and distraction forces that will act on the anterior column.

#### Type A2.1

Spinous processes and facet joints are fractured due to vertical compression. Incomplete and non-displaced lamina fractures and facet joints on multiple levels may be seen (Fig. 2.1). The anterior and middle columns are not affected. Due to incomplete or non-displaced lamina fractures, neurological injury is unlikely. This is a stable injury, and conservative treatment is recommended.

**Fig. 2:**
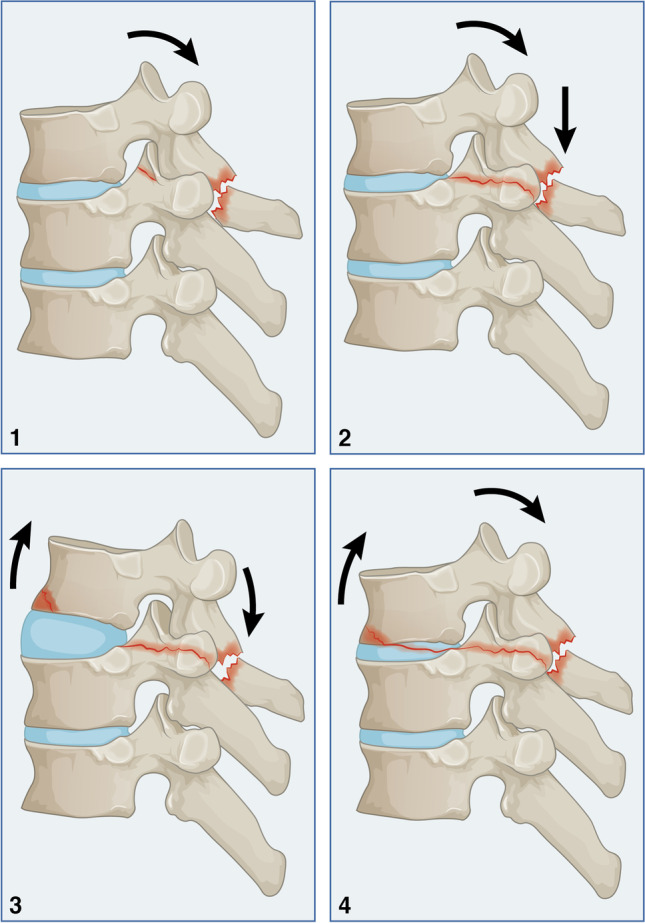
Compression-extension injury (Type A2)

#### Type A2.2

This injury is characterized by the involvement of the costovertebral complex, which can be unilateral or bilateral (Fig. 2.2). Displacement of bony fragments is rare because of intact anterior and middle columns. As a result of that, neurological injury is not expected. The involvement of the fourth column may bear a potential instability. In that case, surgical treatment may be preferred.

#### Type A2.3

This injury is characterized by additional involvement of the anterior column. A teardrop fracture of the inferior anterior corner of the proximal vertebral fracture or injury to the anterior longitudinal ligament can be seen. The bony lesion can be seen as a corner fracture of the vertebral body or a separated bony chip that can be appreciated in computer tomography. Typically, the disc and middle column are not affected. The vertebral height and spinal canal are maintained, and neurological injury is not expected. However, a potential instability exists due to the involvement of two vertebral columns, and the fourth column needs to be carefully evaluated. Therefore, spinal stabilisation may be required.

#### Type A2.4

This injury is characterized by additional discal involvement. The fracture line extends from anterior to posterior through the disc, resulting in three-column affection (Fig. 2.4). Due to the absence of a rotational component during the mechanism of injury, vertebral translation in both sagittal and coronal planes is not seen. However, during an abrupt mechanism of injury, a neurological injury may occur to the spinal cord or nerve root. The fourth column may fail indirectly due to hyperextension. This is an unstable fracture, and surgical stabilisation is preferred.

### Distraction injury (Type B)

In this category, a distraction force acts primarily on the posterior column. At the same time, a secondarily compression force is exerted on the vertebral body. The distraction injury occurs with forward flexion proximally as a result of thoracic kyphosis [17].

#### Type B1

This injury is characterized by rupture of the interspinous ligaments, which may present as increased interspinous distance or fracture of the spinous process that may occur at single or multiple levels (Fig. 3.1). This type of injury is stable and is treated conservatively.

**Fig. 3:**
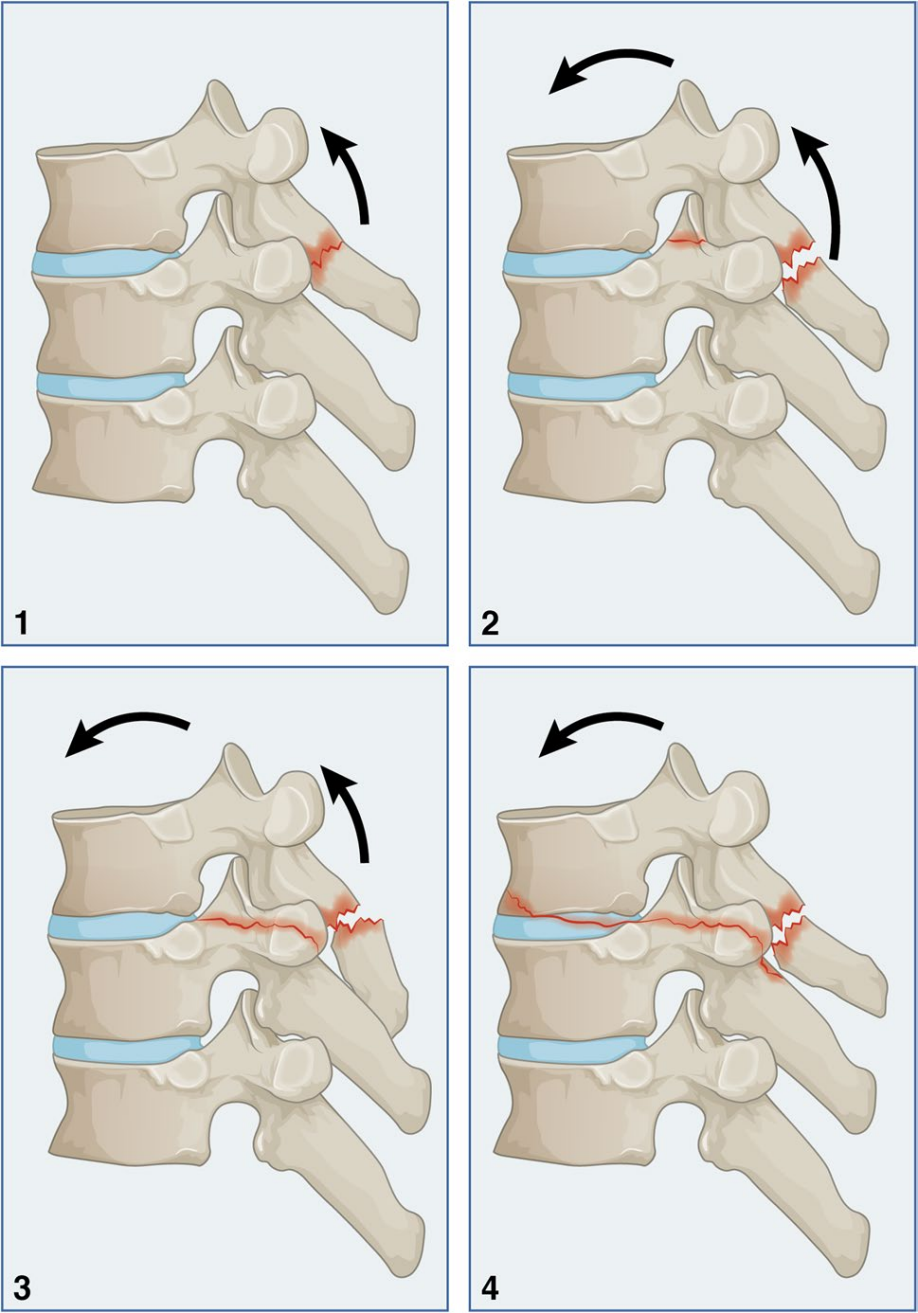
Distraction injury (Type B)

#### Type B2

A non-displaced or minimally displaced laminar fracture or a diastasis of the facet joint can be observed additionally. However, the costovertebral complex and anterior and middle vertebral columns are unaffected (Fig. 3.2). Due to that, neurological injury is not expected, and it is considered a stable fracture and conservative treatment is recommended.

#### Type B3

In addition, to fracture of the costovertebral complex, which may be unilateral or bilateral, pedicle fracture is a characteristic feature of this injury (Fig. 3.3). Both anterior and middle columns are unaffected, and no vertebral translation can be seen in the sagittal plane. As a result of that, neurological injury is unlikely. The instability of the fourth column may carry a potential instability, and surgical stabilisation is favoured.

#### Type B4

The discal extension is a characteristic in a posterior-anterior direction. Anteriorly, the fracture line may extend to the anterior discal end or the anterior vertebral body, leading to a teardrop lesion (Fig. 3.4). A minimal vertebral translation can be seen, and neurological injury may occur. Failure of the fourth column may occur due to an indirect mechanism via further hyperextension. This is a three-column injury, and surgical stabilisation is recommended.

### Rotational injury (Type C)

The vertebral column is vulnerable to rotational and shear forces [18, 19]. The combination of forces can cause discs, joints, and ligaments to be injured [20]. Rotational injuries may occur due to flexion or extension, resulting in different fracture patterns.

### Rotational flexion injury (Type C1)

#### Type C1.1

The rotational force results in a fracture in the posterior corner of the vertebral body or the pedicles (Fig. 4.1A and B). The preferential site of fracture seems to be related to the rotational angle. When combined with flexion, a high rotational angle will likely shift the acting forces to a more posterior location, i.e., the pedicle. On the other hand, at a small rotational angle the forces are likely to concentrate on the posterior corner of the vertebral body. The anterior and middle columns are unaffected, and no widening of the spinal canal can be seen. This injury is stable in the absence of fourth-column instability.

**Fig. 4:**
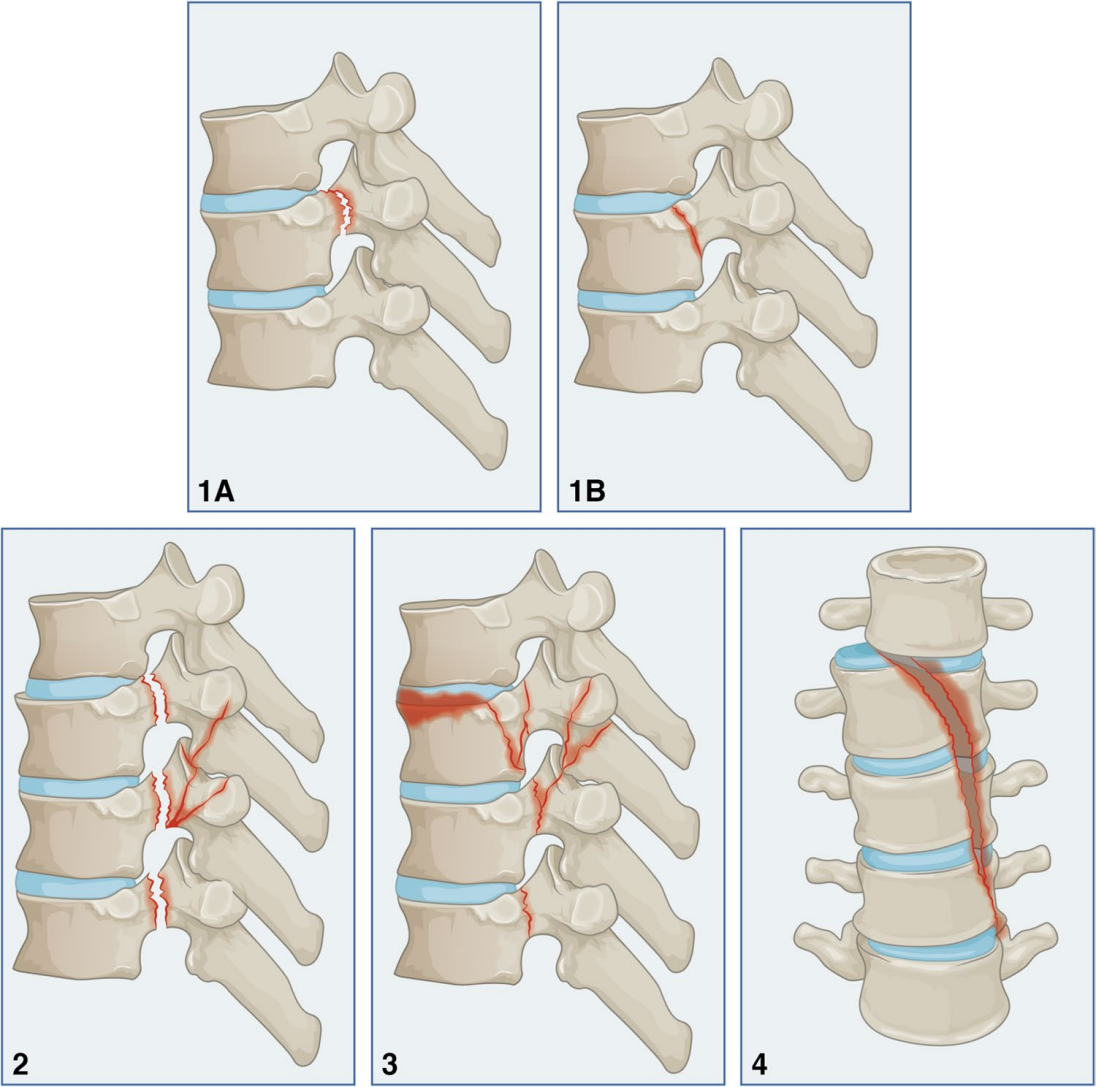
Rotational-flexion injury (Type C1)

#### Type C1.2

The fractures extend to multiple vertebral levels proximally. Multiple fracture pedicles and facet joints can be identified (Fig. 4.2). This injury pattern leads to the posterior column’s separation and the spinal canal’s widening. The widening of the spinal canal is seen over multiple levels, which may serve as neuroprotective phenomena, consequently neurological injury is unlikely. Nonetheless, potential instability may exist since floating of the posterior structures may lead to a secondary neurological injury, and surgical stabilisation may be considered.

#### Type C1.3

The vertebral body fracture occurs as a burst or wedging pattern or coronal split leading to a sliced vertebra involving more than two vertebras (Fig. 4.3). There is no subluxation on the coronal plane; however, a neurological injury may occur due to a bony retropulsion of the vertebral body. This is a three-column injury, and surgical fixation is recommended.

#### Type C1.4

This injury is characterised by a lateral translation of the distal vertebras in the coronal plan (Fig. 4.4). Neurological injury is expected because of the complete discontinuation of the vertebral column coronally. Therefore, this is a complete instability injury, and surgical stabilisation is recommended to restore spinal alignment.

### Rotational extension injury (Type C2)

#### Type C2.1

Combined extension and vertical compression on the posterior column led to spinous process fractures at single or multiple vertebral levels (Fig. 5.1). The facet joints are unaffected at this stage due to the rotational component, which leads to distal propagation of the fracture line distally toward the costovertebral complex, which is intact in this subcategory. Since both the facet joint and costovertebral complex are intact, neurological injury is not expected. This is stable and conservative treatment is recommended.

**Fig. 5:**
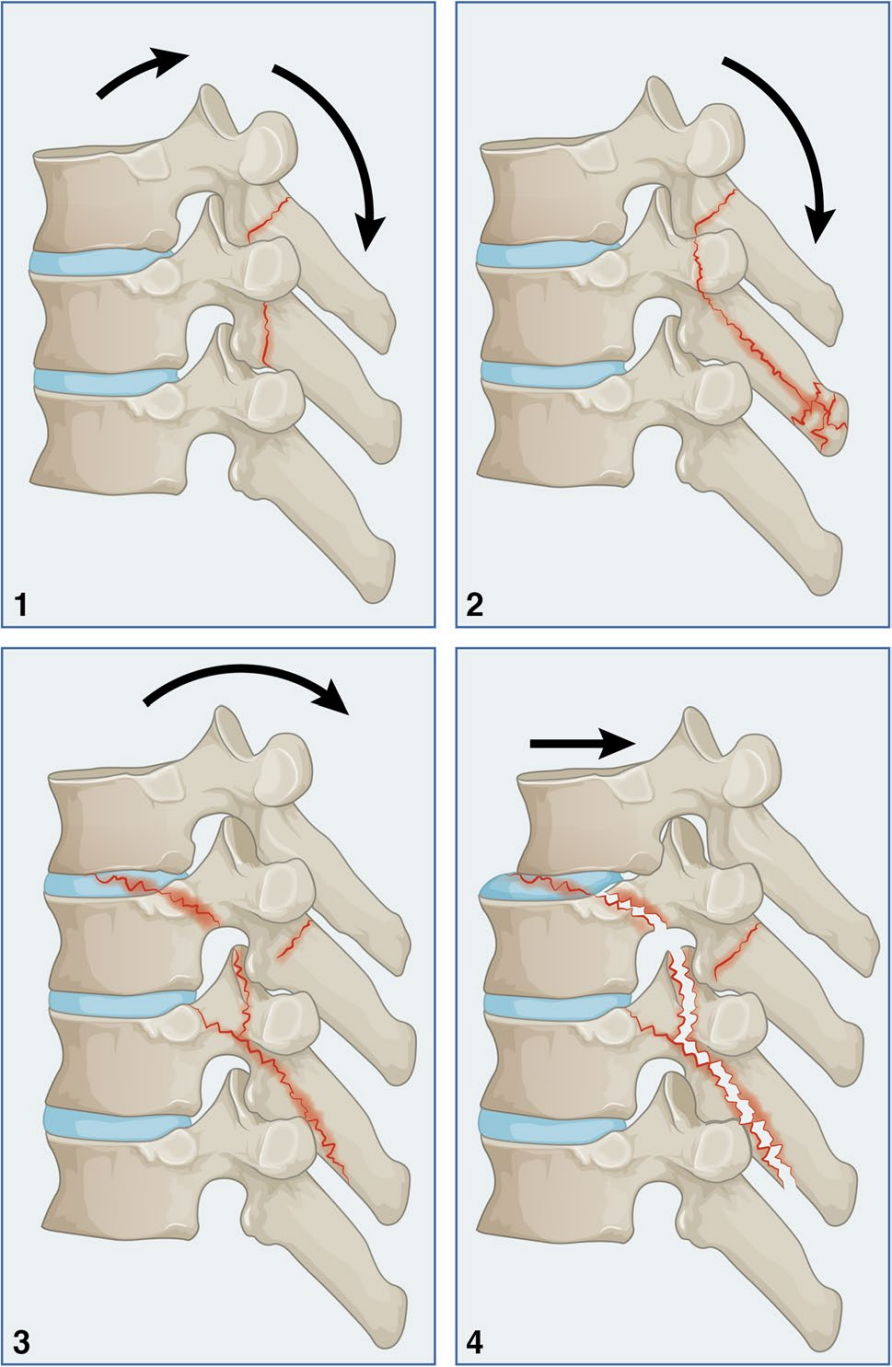
Rotational extension injury (Type C2)

#### Type C2.2

This injury is characterized by the distal extension of the fracture line caudally involving the costovertebral complex and lamina, which may be detected as a non-displaced fracture (Fig. 5.2). Facet joint involvement may occur; however, pedicle involvement is not seen due to caudal fracture line extension. Intact facet joints and pedicles may preclude neurological injury that serves as a neuroprotective element. Careful evaluation of the fourth column is necessary because of the involvement of the costovertebral joints, especially in bilateral involvement. Potentially instability may exist in the presence of instability in the fourth column, and surgical stabilisation may be favoured.

#### Type C2.3

Due to further hyperextension, pedicle fractures at multiple vertebral levels occur cranially. In addition, discal involvement is in the form of a corner fracture of the posterior wall vertebral body or involvement of the entire disc. The vertebral translation is not seen at the time of evaluation. However, this complex injury may occur with transit translation during the mechanism of the injury with spontaneous relocation (Fig. 5.3). Hence, neurological injury is likely. Due to three-column injury, this is unstable, and surgical stabilisation is recommended.

#### Type C2.4

Posterior subluxation of the proximal vertebral in the sagittal plane is noticed with a floating lamina (Fig. 5.4). This injury is unstable, and surgical stabilisation is recommended to restore spinal alignment.

## Discussion

The proposed classification is developed for fractures of the thoracic spine. To our knowledge, it is the first classification focused solely on the thoracic spine. The classification incorporates anatomical and biomechanical characteristics that distinguish the thoracic spine from other spinal regions. A mechanistic platform conceptualised the classification, combining movement and forces acting on the spine. Various fracture patterns are explained according to the type and magnitude. Within the subdivisions of the classification, fracture severity is presented hierarchically by considering the degree of movement and the magnitude of the load.

The classification is an evolution of the original work of Holdsworth [15], who considered movement a form of violence leading to a spine fracture. He proposed a classification based on two-column model, incorporating: flexion, flexion and rotation, extension, and compression. Other researchers support the notion that spine fracture results from exaggerated movement [21, 22]. On the other hand, commonly used classifications in other spine regions were established on common forces acting on the spine, such as compression, distraction, and torsion [8, 11]. However, both movement and force are closely related. Any change in the motion is due to the force acting on the body, and any unequal forces will result in deformation or displacement. This can be explained by Newton’s law [23]. Consequently, both motion and force are tight up in a deformation mechanism.

As a result, the classification provides a new mechanistic approach by considering both movement and force acting on the spine. The advantages of this two-dimensional relationship are numerous. As a result, it represents a dynamic relationship determined by direction and magnitude that explains most fracture patterns. Second, it provides a comprehensive explanation of the fracture mechanism. Third, it might also explain indirect rib cage injuries.

Berge [16] introduced the concept of the fourth column. According to him, the sternum and the ribs represent an additional column of stability for the thoracic spine. He stressed that “no matter how minimal the wedge fracture in the thoracic spine may appear, there is potential for instability if there is a concomitant displacement of the sternum”. In the following years, other scholars supported the concept of the fourth column [18, 19].

The fourth column of the thoracic spine includes the sternum and ribs, and their contribution to the thoracic spine stability was demonstrated in biomechanical analyses [24–31]. Despite that, there is still a lack of data regarding the effect of concomitant rib cage injuries in the context of thoracic spine injuries [32]. Nevertheless, the instability of each component of the rib cage has been reported separately in the literature, and it is feasible to establish a deposition.

Sternal fractures can occur through both direct and indirect mechanisms. Interestingly, indirect sternal fractures have been reported in thoracic spine fractures [16, 33, 34]. Based on these reports, the sternum may be fractured indirectly due to distraction, compression-hyperflexion, and hyperextension of the thoracic spine. Consequently, during the development of the classification, injury to the fourth column is incorporated from two perspectives: firstly, as a modifier to indicate potential instability in a direct mechanism and, secondly, as a consequence of indirect mechanism involvement. The indirect mechanisms of sternal fracture are characterised by a common prerequisite — failure of the posterior column. By doing so, the sternum could act as a tension band to stabilise the thoracic spine. The tension band theory of the sternum can be supported by the fact that a 40% reduction in thoracic spine stability has been reported following sternal fractures [29] and sternal release [35].

The instability of the fourth column may be represented by displaced fractures of the sternum’s body and the manubrium. In addition, consecutive multiple rib fractures and a flail chest should not be ignored. Several variations of the flail chest exist, such as the combination of sternal fractures with rib fractures or the fracture of multiple bilateral sternal costal attachments [36]. Although no quantification studies have investigated their impact on thoracic spine stability, they should be carefully evaluated individually since the instability of one may lead to the instability of the others [19].

Another concept introduced in the classification is the posterior bony complex. The laminae are short, thick, and broad in the thoracic spine. The thoracic laminae and spinous processes are directed backwards and downward, overlapping like roof tiles [37]. Unlike lumbar spine, the bony “complex” geometry seen posteriorly in the rib cage is an integrated structure that acts as opposed to excessive movement.

It appears that the posterior bony complex of the thoracic spine can be justified based on the evidence available. In support of this rationale, biomechanical studies have been conducted on the role of posterior elements in the thoracic spine. Evidence indicates that a sequential resection of the “posterior bony complex” has led to a progressive increase in movement in every plane of movement [25, 27]. Adapting the rationale of the posterior bony complex in the thoracic spine seems coherent.

The proposed classification accommodates several advantages, such as simplicity and practicality, that make this classification helpful in daily practice. The dynamic relationship between movement and force provides a better understanding of the fracture mechanism. Finally, incorporating the fourth column will strengthen the indication for surgical management. Indeed, investigating its validity and reliability will further strengthen its proposition. Even though the fourth column is included, many types of injuries can occur, and a scoring system might help to improve the prognostic value of the proposed classification. For its adaptation in clinical practice, collaborative efforts are needed to reach a consensus.

## Conclusion

While spine fracture classification has evolved significantly over a century, thoracic spine fractures remain understudied. This paper presents a unique classification developed specifically for the thoracic spine. Adapting both the anatomical and biomechanical characteristics of the thoracic spine is supported by growing evidence. Thus, it provides additional insight into the instability of the thoracic spine during direct and indirect injury to the fourth column. As a tool for clinical practice, it has been designed in a simple format that is essential for daily use and communication. The need for additional research in the future cannot be overstated. Its validity and reliability must be evaluated to determine its constructive and predictive merit.

## Data Availability

Not applicable.
